# *Bifidobacterium animalis subsp lactis* HN019 presents antimicrobial potential against periodontopathogens and modulates the immunological response of oral mucosa in periodontitis patients

**DOI:** 10.1371/journal.pone.0238425

**Published:** 2020-09-22

**Authors:** Marcos M. Invernici, Flávia A. C. Furlaneto, Sérgio L. Salvador, Arthur C. Ouwehand, Seppo Salminen, Anastasia Mantziari, Gabriel Vinderola, Edilson Ervolino, Sandro Isaías Santana, Pedro Henrique Felix Silva, Michel R. Messora

**Affiliations:** 1 Department of Oral and Maxillofacial Surgery and Periodontology, School of Dentistry of Ribeirão Preto, University of São Paulo – USP, Ribeirão Preto, São Paulo, Brazil; 2 Department of Clinical Analyses, School of Pharmaceutical Sciences of Ribeirão Preto, University of São Paulo – USP, Ribeirão Preto, São Paulo, Brazil; 3 Dupont, Nutrition and Health, Kantvik, Finland; 4 Functional Foods Forum, University of Turku, Turku, Finland; 5 Instituto de Lactología Industrial (UNL-CONICET), National University of the Litoral, Santa Fe, Argentina; 6 Division of Histology, Department of Basic Sciences, Dental School of Araçatuba, São Paulo State University, São Paulo, Brazil; University of the Pacific, UNITED STATES

## Abstract

**Objective:**

To evaluate the effects of *Bifidobacterium animalis* subsp. *lactis* HN019 (HN019) on clinical periodontal parameters (plaque accumulation and gingival bleeding), on immunocompetence of gingival tissues [expression of beta-defensin (BD)-3, toll-like receptor 4 (TLR4), cluster of differentiation(CD)-57 and CD-4], and on immunological properties of saliva (IgA levels) in non-surgical periodontal therapy in generalized chronic periodontitis (GCP) patients. Adhesion to buccal epithelial cells (BEC) and the antimicrobial properties of HN019 were also investigated.

**Materials and methods:**

Thirty patients were recruited and monitored clinically at baseline (before scaling and root planing—SRP) and after 30 and 90 days. Patients were randomly assigned to Test (SRP+Probiotic, n = 15) or Control (SRP+Placebo, n = 15) group. Probiotic lozenges were used for 30 days. Gingival tissues and saliva were immunologically analyzed. The adhesion of HN019 with or without *Porphyromonas gingivalis* in BEC and its antimicrobial properties were investigated in *in vitro* assays. Data were statistically analyzed (*p<0*.05).

**Results:**

Test group presented lower plaque index (30 days) and lower marginal gingival bleeding (90 days) when compared with Control group. Higher BD-3, TLR4 and CD-4 expressions were observed in gingival tissues in Test group than in Control group. HN019 reduced the adhesion of *P*. *gingivalis* to BEC and showed antimicrobial potential against periodontopathogens.

**Conclusion:**

Immunological and antimicrobial properties of *B*. *lactis* HN019 make it a potential probiotic to be used in non-surgical periodontal therapy of patients with GCP.

**Clinical relevance:**

*B*. *lactis* HN019 may be a potential probiotic to improve the effects of non-surgical periodontal therapy.

**Name of the registry and registration number** (ClinicalTrials.gov): “Effects of probiotic therapy in the treatment of periodontitis”—NCT03408548.

## Introduction

In periodontal diseases, the first triggering mechanism is dental plaque accumulation as a result of poor oral hygiene [1]. Some studies have suggested that several periodontal pathogens may colonize supragingival biofilm, which can contribute to subgingival recolonization of recently treated sites [2–5]. This finding has led to strict professional plaque control (PPC) as part of the periodontal treatment [6, 7], but with the inconvenience that it is difficult to ensure that patients return to the dental office frequently for long periods. Maintenance of gingival health after non-surgical periodontal therapy (NSPT) could prevent the increase of gingival crevicular fluid and, consequently, the augmentation of proteins that act as a source of nutrients for periodontopathogens [1]. In fact, it has been demonstrated that one promising strategy for the control and treatment of periodontal diseases is the modulation of the host inflammatory response, since it is evident that some keystone pathogens are attracted by inflammation. Therefore, the control of inflammation is of paramount importance for managing the infection [8].

In this context, a possible adjuvant therapy for the prevention of gingival inflammation and dental plaque accumulation in non-surgical periodontal treatment is the use of probiotics. Probiotics are defined as “live microorganisms which when administered in adequate amounts confer a health benefit on the host” [9]. Probiotics might play a possible role in oral health by suppressing and displacing harmful bacteria and indirectly by producing immunomodulatory effects [10].

A number of *in vivo* studies have focused on the role of probiotics in the prevention and treatment of periodontal diseases [11–17]. It has been shown that probiotics are useful in reducing gingival inflammation [11–15] and plaque accumulation [12, 13], improving periodontal health [11, 16, 17] and reducing proinflammatory cytokines in patients with gingivitis [14] and periodontitis [18].

To be effective against oral infections, probiotic bacteria need to adhere to the oral mucosa and to dental tissues as part of the biofilm and compete with the growth of dental pathogens. In fact, it has been demonstrated that some probiotic bacteria can interfere both quantitative and qualitatively with the oral biofilm [19–21]. Haukioja et al. 2006 demonstrated a trend for two *Bifidobacterium* strains to bind to buccal cells and intestinal mucus [22]. This ability suggests that probiotics might modulate innate and adaptive immune defenses by releasing soluble factors which can trigger signaling cascades in epithelial cells [23, 24]. In short, different immune components actively kill, inhibit, and agglutinate microbes [lysozyme, defensins, histatins, immunoglobulin (Ig) A], deprive them of iron (lactoferrin), prevent their adhesion (IgA, IgG, IgM), or act as opsonins (complement, IgG, IgM) that increase phagocytosis by immune cells [25].

Bifidobacteria occur naturally in the oral cavity [26] and are among the most predominant anaerobic bacteria within the intestinal lumen. A body of evidence suggests that bifidobacteria play a critical role in maintaining the normal balance of the gut microbiota [27], and a number of probiotic-induced benefits to general health have been proposed. The possible impact on oral health has been poorly explored.

Kuru et al. (2017) demonstrated that the use of a probiotic yogurt supplemented with *B*. *animalis* could have a positive effect on plaque accumulation and on gingival inflammation after abstinence from oral hygiene practices [19]. Ricoldi et al. (2017) observed that *Bifidobacterium animalis* subsp. *lactis* (*B*. *lactis*) HN019 increased the expression of anti-inflammatory cytokines and reduced the expression of proinflammatory cytokines in rats with experimental periodontitis [28]. Oliveira et al. (2017) verified greater osteoprotegerin and beta-defensins (BD) expressions in periodontal tissues of rats with experimental periodontitis treated with *B*. *lactis* HN019 when compared with untreated rats [29].

A recent study demonstrated the effects of *B*. *lactis* HN019 as an adjunct to non-surgical therapy in generalized chronic periodontitis (GCP) patients [30]. The patients treated with probiotic experienced superior results regarding decrease in probing pocket depth and clinical attachment gain. Furthermore, they demonstrated fewer periodontal pathogens of red and orange complexes and reduced proinflammatory cytokine levels in gingival crevicular fluid when compared with patients using placebo.

However, to the authors’ knowledge, no studies have examined the mechanisms of action, including immunomodulatory properties, of *Bifidobacterium*-supplemented probiotics in the oral mucosa of patients with periodontitis. Also, no evaluations regarding the adhesion of *B*. *lactis* HN019 to buccal epithelial cells and *B*. *lactis* HN019 antimicrobial activity have been performed. The aim of this study was to evaluate the effects of *B*. *lactis* HN019 on clinical periodontal parameters (plaque accumulation and gingival bleeding), on the immunocompetence of gingival tissues [expression of BD-3, Toll-like receptor 4 (TLR4), cluster of differentiation (CD)-57 and CD-4], and on immunological properties of saliva (IgA levels) in non-surgical periodontal therapy in GCP patients. Adhesion to buccal epithelial cells and antimicrobial properties of *B*. *lactis* HN019 were also investigated.

## Materials and methods

### Patient population

This study includes part of the sample of patients from the study by Invernici et al. [30]. Thirty patients were selected from the population referred to the Periodontal Clinic at the School of Dentistry of Ribeirão Preto–University of São Paulo (FORP-USP, Ribeirão Preto, SP, Brazil). All eligible patients were thoroughly informed of the nature and potential risks and benefits of their participation in the study and signed an informed consent form. The study protocol was reviewed and approved by the Research Ethics Committee of FORP-USP (protocol number: 06278012.1.0000.5419), and registered in ClinicalTrials.gov (NCT03408548). The research was conducted in full accordance with ethical principles, including the World Medical Association Declaration of Helsinki (version 2008) and additional requirements.

### Inclusion and exclusion criteria

All patients were diagnosed with GCP according to the 1999 classification of the American Academy of Periodontology [31]. The inclusion criteria were: (1) age over 30 years, (2) 30% or more of the sites with probing pocket depth (PPD) ≥ 4 mm and clinical attachment level (CAL) ≥ 4 mm, (4) presence of bleeding on probing (BOP) and a minimum of five teeth with at least one site with CAL and PPD ≥ 5 mm. All patients had to be in good general health. The exclusion criteria were: (1) cause-related periodontal therapy in the previous 6 months, (2) systemic and topic antimicrobial therapy (i.g. antibacterials, antifungals, antivirals and antiseptics) in the previous 6 months, (3) systemic conditions that could influence the progression of periodontitis or treatment response (i.e., diabetes mellitus, immunological disorders), (4) pregnancy, (5) smoking, (6) extensive prosthetic appliances, (7) need of prophylactic antibiotic therapy for routine dental procedures, and (8) long-term administration of anti-inflammatory medications.

### Experimental design, allocation concealment, and treatment protocol

**Fig 1:**
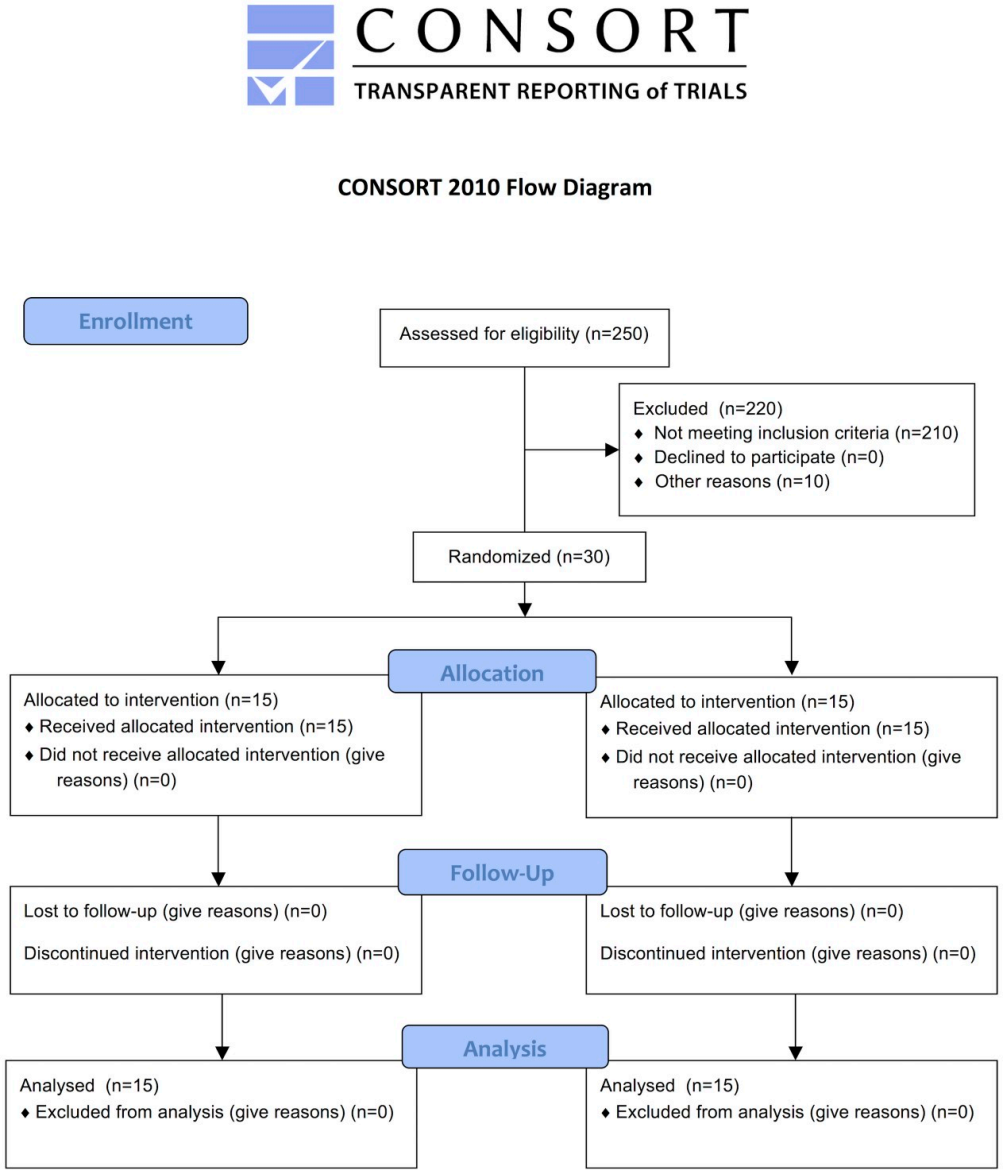
Flowchart of the study design.

According to a random numeric table generated by a computer software, the study coordinator (M.R.M.) allocated each patient to one of the following groups: Control (Scaling and root planing–SRP + placebo; 15 patients) or Test (SRP + probiotic therapy, 15 patients; Fig 1). Before the study began, the selected individuals were identified by a numeric code that designated the experimental group to which they belonged. The study coordinator (M.R.M.) broke the code only after conducting the statistical analysis of the experimental data.

The patients received lozenges containing 10 mg of probiotic (Test group) or placebo (Control group). In the Test group, the lozenges had 10^9^ colony-forming units (CFUs) of *B*. *lactis* HN019 (HOWARU^®^ Bifido LYO 40 DCU-S, DuPont^™^ Danisco^®^ Sweeteners Oy, Kantvik, Finland). The same pharmacy prepared identical probiotic and placebo lozenges (i.e., same appearance, taste, and color). Identical plastic bottles containing the probiotic/placebos were sent to the study coordinator (M.R.M.), who wrote the number code of each patient on each bottle, according to the therapy to which they were assigned. The coded bottles were given to the examiner (M.S.M.S.), who was blinded to the content of the lozenges and distributed them to the patients. In addition, the patients were also blinded to the content of the lozenges and treatment assignment during the study.

Seven days prior to NSPT, all patients received supragingival plaque control and oral hygiene instructions. Within 24 h, a specialist in periodontics (M.M.I.), who was blinded to the experimental groups, performed supragingival and subgingival SRP on all teeth with periodontal involvement, using hand (Gracey Curettes, Hu-Friedy, Chicago, IL, USA) and ultrasonic instruments. The patients were instructed (immediately after SRP) to take one lozenge twice a day (in the morning and before bedtime) for 30 days. They were also instructed not to take any other probiotic product during the study. The patients received 14 lozenges (placebo or probiotic) per week. At the end of each week, they had to be seen at the FORP-USP Periodontal Clinic. During their visit, they should bring the packs of lozenges taken during the week and then they received new lozenges for the subsequent week. At this visit, they were asked to a questionnaire about their perception of any side effect observed during the consumption of the dietary supplement. One research assistant (P.H.F.S.) conducted these procedures and monitored the patient’s compliance with medication dosage. This assistant was not examiner or operator in this study.

All patients received immunological and clinical monitoring at baseline, at 30 days, and at 90 days. The evaluations (pre- and post-intervention) were conducted by a single trained and calibrated examiner (M.S.M.S.), who was blinded to the experimental groups.

### Examiner calibration

The Kappa coefficient was used to evaluate examiner calibration regarding the collection of clinical periodontal parameters for calculation of intra-examiner agreement. Ten patients with gingivitis (with both bleeding and non-bleeding on probing) not included in the study were evaluated by the examiner on two separate occasions 48 h apart. Plaque index (PI) and bleeding on marginal probing (BOMP) were measured. Calibration was accepted if measurements at baseline and at 48 h were consistent in 90% of the measurements.

### Clinical measurements

PI was employed to assess the patients’ oral hygiene status. The BOMP value was described 30 s after gently running a periodontal probe (PCPUNC156, Hu-Friedy, Chicago, IL, USA) into the gingival sulcus. PI and BOMP were scored as absent or present visual plaque and bleeding (0 or 1, respectively). BOMP and PI were measured at four sites per tooth at baseline, at 30 days, and at 90 days.

### Analysis of salivary immunoglobulin A (IgA) levels

At baseline, and also at 30 and 90 days after NSPT, unstimulated saliva was collected in the morning, after a fasting period of at least 8 h. The patients were instructed not to move the tongue, cheeks, or lips during saliva collection. Two minutes after instructions, the patients spat into the spit sink, thus eliminating initial residues. After that, collection began. Three 3-minute cycles were run, during which the patients kept the saliva in the oral cavity. At the end of that period, the patients’ residual saliva was collected into a 50-mL polypropylene Falcon tube (Corning Inc, Corning, NY, USA). The procedure was repeated twice and total collection time was 9 min. The saliva samples were then transferred into two graduated Eppendorf tubes using a handheld micropipette. Each tube with 1 mL of saliva was stored at -80 °C. IgA levels (mg/dL) were measured in a clinical analysis laboratory by nephelometry at 1.59 to 41.45 mg/dL.

### Gingival biopsies and immunohistochemical analysis

At baseline, and also at 30 days after NSPT, samples of gingival tissues (epithelial and connective tissues) were harvested from a healthy site (PPD < 3 mm) and from a diseased site (PPD > 5 mm) of each patient. Gingival biopsies were obtained through internal bevel incisions including supracrestal epithelial and connective tissues. Gingival biopsies were embedded in paraffin. Serial sections (4 μm) were performed for visualization of epithelial and connective tissues. Histological sections were deparaffinized in xylol and hydrated in a decreasing ethanol series (100°- 100°- 100°- 90°- 70° GL). Antigen retrieval was performed by immersion of the histological slides in Diva Decloaker buffer (Biocare Medical, Inc., Concord, CA, USA), in a pressurized chamber (Decloaking Chamber, Biocare Medical, Inc., Concord, CA, USA) at 95° C for 10 min. The samples were rinsed with phosphate-buffered saline (PBS) 0.1M - pH 7.4 between each immunohistochemical step. The histological sections were immersed in 3% hydrogen peroxide for 1 h and in 1% bovine serum albumin for 12 h to block endogenous peroxidase activity and nonspecific sites, respectively. Histological slides with samples from all experimental groups were categorized into four lots. Each lot was incubated for 24 h using the following primary antibodies: anti-BD-3 (Santa Cruz Biotechnology, Inc., Santa Cruz, CA, USA), anti-TLR4 (Santa Cruz Biotechnology, CA, USA), anti-CD-57 (Santa Cruz Biotechnology, CA, USA) or anti-CD-4 (Santa Cruz Biotechnology, CA, USA). Universal LSAB^™^+ Kit/HRP (Dako North America, Inc., Carpinteria CA, USA) was used in the subsequent stages. The histological sections were incubated in biotinylated secondary antibody for 2 h and treated with a streptavidin–horseradish peroxidase conjugate for 1 h. 3,3’- diaminobenzidine tetrahydrochloride hydrate (Liquid DAB+, Dako North America, Inc., Carpinteria CA, USA) was used as chromogen, and the histological sections were counterstained with Harris hematoxylin. As negative control, the specimens were subjected to the procedures described earlier, without primary antibodies.

Histological sections were analyzed semiquantitatively by a researcher who was blinded to the experimental groups. The immunolabeled areas were standardized using 250x magnification in a 400 μm x 600 μm area at the center of the histological section. The long axis of this rectangle was perpendicular to an imaginary line at the interface between the epithelial and connective tissues. The rectangle was positioned in such a way that its upper half contained predominantly epithelial tissue, whereas its lower half contained predominantly connective tissue. Immunolabeling was defined as a brownish precipitate in cells and/or in the extracellular matrix captured by a digital camera coupled to a light microscope and later saved in a computer file. Immunolabeling scores were used as follows: 0 –no immunolabeling (no immunoreactivity in the area); score 1 –extremely low immunoreactivity (around 1⁄5 of the area was immunoreactive); score 2 –low immunoreactivity (around 2⁄5 of the area were immunoreactive); score 3 –moderate immunoreactivity (around 3⁄5 of the area were immunoreactive); score 4 –high immunoreactivity (around 4⁄5 of the area were immunoreactive); score 5 –extremely high immunoreactivity (almost the whole area was immunoreactive).

### *In vitro* assay of the adhesion of *B*. *lactis* HN019 and *Porphyromonas gingivalis* to buccal epithelial cells (BEC)

The assay was performed as previously described [22], with some modifications as follows: BEC were collected from one healthy male volunteer. Cells were washed and suspended in buffered KCl to obtain an optical density (OD)600 of 0.500. Bacterial suspensions were made from overnight cultures in buffered KCl to obtain an OD600 of 0.1 [= 2 x 10^7^ colony forming units (CFU)/mL]. Equal amounts of BEC and bacterial suspensions were incubated for 60 min. Control cells were treated with buffer only. After washing, the cells with adherent bacteria were stained with crystal violet and safranin solutions. All bacteria (indigenous bacteria, *B*. *lactis* HN019, and *P*. *gingivalis*—ATCC 33277) adhered to BEC were counted under a light microscope. Thirty cells were counted from each sample. All bacterial strains were tested in parallel and the experiments were repeated twice.

### *In vitro* analysis of the antimicrobial activity of *B*. *lactis* HN019

The *in vitro* antimicrobial activity of *B*. *lactis* HN019 was assessed against the following periodontopathogens using the agar diffusion method, as described by Zhu et al. [32]: *P*. *gingivalis* (W83), *Prevotella intermedia* (ATCC 25611), *Fusobacterium nucleatum* (ATCC 25586), and *Aggregatibacter actinomycetemcomitans* (ATCC 33393). For that purpose, 200-μL aliquots (10^9^ CFU/mL) of *B*. *lactis* HN019, previously grown on MRSA (Difco Laboratories, Detroit, MI, USA), were inoculated into 15-mm wells in Tryptic soy agar—TSA (Difco), supplemented with 5 μg/mL of hemin, 1 μg/mL of menadione, and 5% of defibrinated sheep blood, previously seeded (1.5 x 10^8^ CFU/mL) with indicator microorganisms. After pre-incubation for 30 min at room temperature, TSA plates were incubated at 37° C for 72 h under anaerobic (BD GasPak^™^ EZ container systems, Becton, Dickinson and Company, Franklin Lakes, NJ, USA) (*P*. *gingivalis*, *P*. *intermedia*, *and F*. *nucleatum*) or microaerophilic (*A*. *actinomycetemcomitans*) conditions. Thereafter, the diameter (mm) of inhibition halos was measured using a digital caliper. Each indicator strain was tested three times in duplicate.

The means and standard deviations of the zones of inhibition observed in the sensitivity of different periodontopathogens to *B*. *lactis* HN019 were calculated.

### Statistical analysis

Each variable was computed per participant and then averaged across patients in both groups. The significance level was set at 5%.

The within-group and between-group differences for i) PI; ii) BOMP; and iii) IgA levels were assessed by repeated-measures analysis of variance (ANOVA) followed by Bonferroni *post-hoc* test and by Student’s t test, respectively.

The relative frequencies of the scores were calculated for the analysis of immunohistochemical data on BD-3, TLR4, CD-57 and CD-4 immunolabeling, at baseline and at 30 days for each experimental group, considering healthy and unhealthy sites separately. Significant differences between groups were determined by the Kruskal-Wallis test, followed by Dunn’s *post-hoc* test.

The between-group and within-group differences for adhesion of the strains to BEC were evaluated by the Kruskal-Wallis test, followed by Dunn’s *post-hoc* test.

## Results

This study started in December 2015 and ended in August 2016. The sample was comprised of 15 patients in the control group (7 female and 8 male) with mean age ± standard deviation (SD) of 47.67±9.49 years and 15 patients in the test group (10 female and 5 male) with mean age ± SD of 47.60±9.97 years. The means of teeth in the mouth ± SD were 24.53 ± 2.97 and 22.86 ± 2.85 in Control and Test groups, respectively. There were no statistically significant differences between these variables. Postoperative healing was uneventful in all cases. No adverse effects of probiotic therapy were observed.

### Clinical monitoring

Table 1 shows mean percentage rates for BOMP and PI. Test group presented lower BOMP at 90 days and lower PI at 30 days when compared to Control group (*p<0*.05).

**Table 1:** Mean % (± SD) of BOMP and PI at baseline, 30 days and 90 days.

Variables	Time point	Treatment groups		Intergroup Comparison
		Test	Control	Student’s t test
				*p* value
BOMP	Baseline	9.17 ± 7.71ª	14.07 ± 7.99^a^	0.0987
	30 days	4.85 ± 5.2^b^	9.38 ± 8.67^b^	0.0938
	90 days	5.92 ± 6.12^a^	12.10 ± 8.19^a^	**0.0267**
PI	Baseline	18.71 ± 12.14ª	22.50 ± 8.54ª	0.2594
	30 days	9.58 ± 5.75^b^	15.33 ± 9.47^b^	**0.0420**
	90 days	18.27 ± 17.11^a^	22.66 ± 9.99^a^	0.2513

BOMP = bleeding on marginal probing; PI = plaque index; SD = standard deviation; Different letters represent significant differences between time points within the same group (repeated measures ANOVA, Bonferroni *post hoc* test, *p*<0.05).

### Analysis of salivary IgA levels

**Fig 2:**
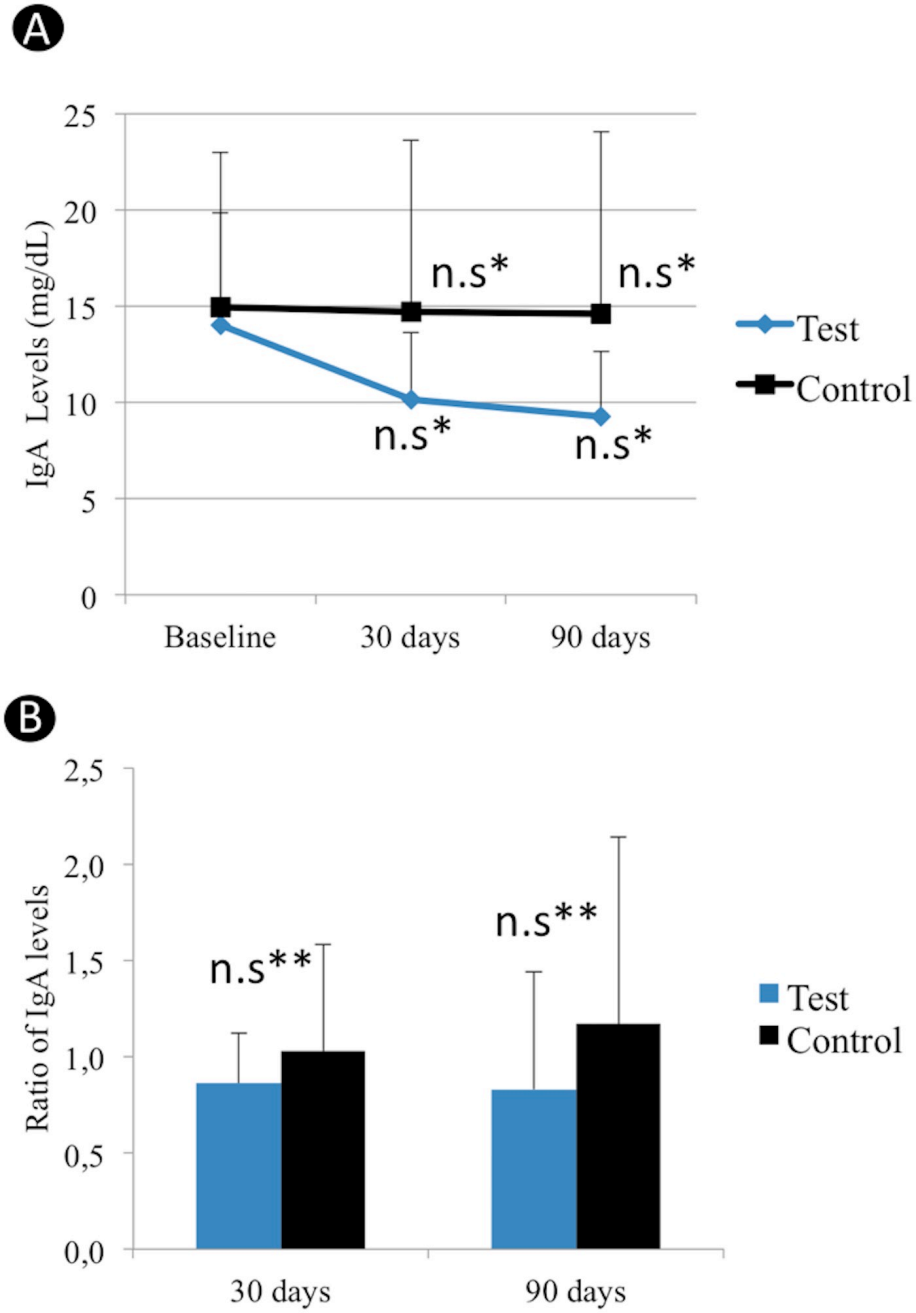
IgA levels (**A**) of Test and Control groups at baseline and at 30 and 90 postoperative days. Mean changes in IgA levels relative to baseline values obtained for the Test and Control groups are shown in (**B**). Ratio of IgA levels = changes in IgA levels in relation to baseline values. n.s* No significant changes were observed in IgA levels at both 30 and 90 days, compared to baseline, for Test and Control groups. n.s** No significant differences between Test and Control groups at both 30 and 90 days.

IgA levels are shown in Fig 2A. No significant changes were observed in IgA levels at 30 and 90 days, compared to baseline, for Test and Control groups (*p*>0.05). Fig 2B shows mean IgA ratios (changes in IgA levels in relation to baseline values) at 30 and 90 days. There were no significant differences between Test and Control groups at 30 and 90 days (*p*>0.05).

### Gingival biopsies and immunohistochemical analysis

**Fig 3:**
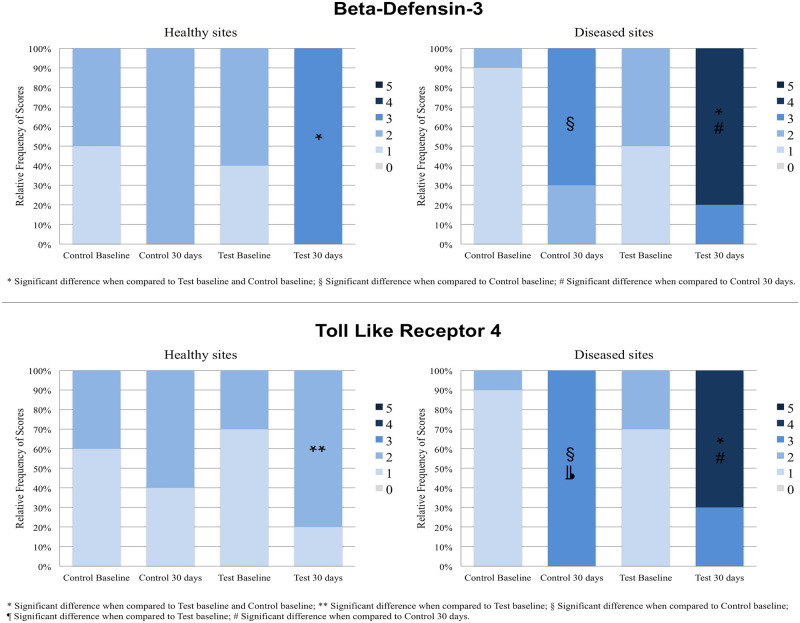
Relative frequency values for BD-3 and TLR4 immunolabeling scores in Test and Control groups at healthy and diseased sites at baseline and at 30 postoperative days, as well as within-group and between-group comparison results. *Significant difference (Kruskal-Wallis, Dunn, *p*<0.05). *Significant difference when compared to Test baseline and Control baseline. **Significant difference when compared to Test baseline. §Significant difference when compared to Control baseline. ¶Significant difference when compared to Test baseline. #Significant difference when compared to Control 30 days.

**Fig 4:**
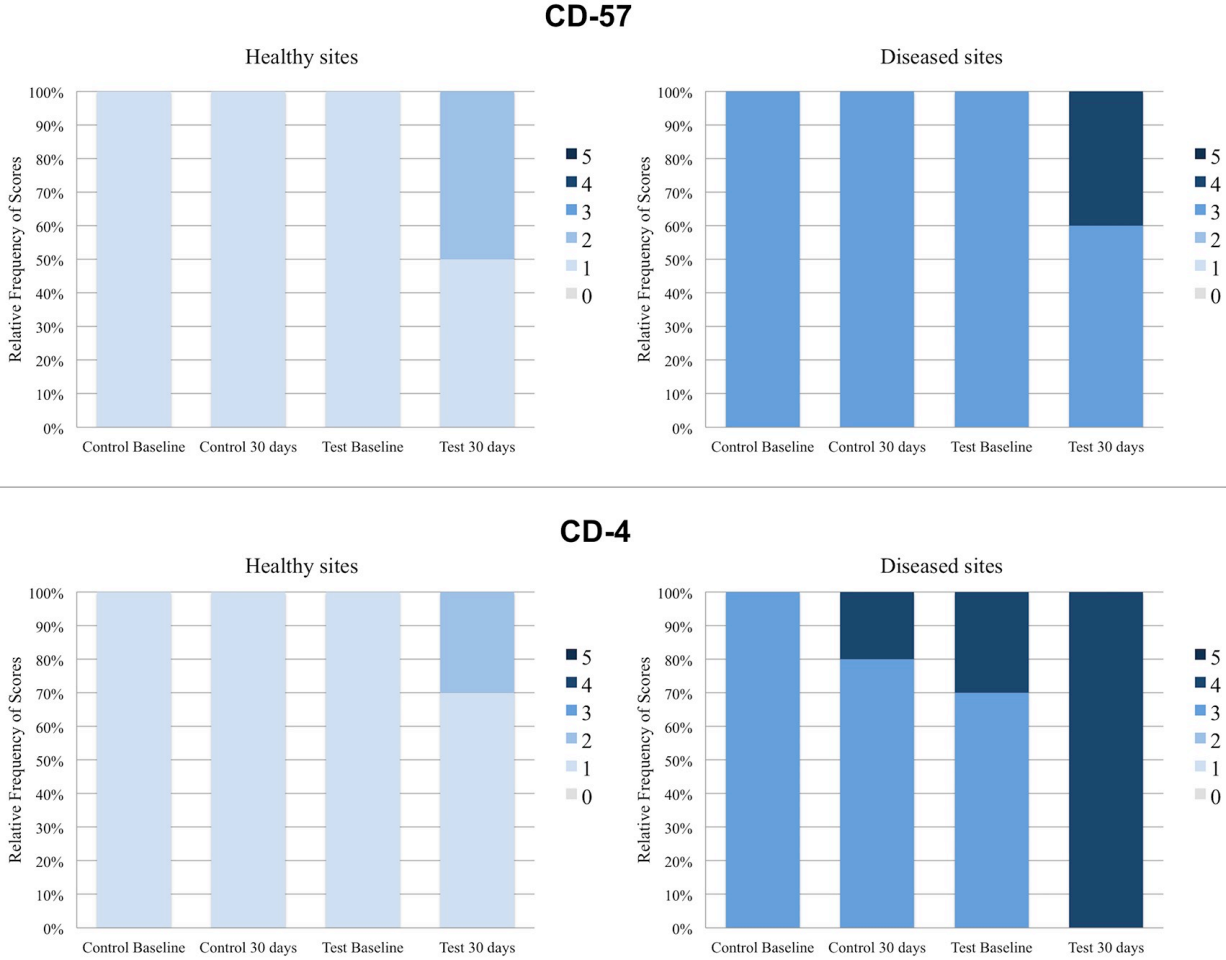
Relative frequency values for CD-57 and CD-4 immunolabeling scores for test and control groups at healthy and diseased sites at baseline and at 30 postoperative days, as well as within-group and between-group comparison results. *Significant difference (Kruskal-Wallis, Dunn, p<0.05). *Significant difference when compared to Test baseline and Control baseline. #Significant difference when compared to Control 30 days.

The relative frequencies of scores of healthy and diseased sites in Control and Test groups before and after probiotic therapy are shown in Figs 3 and 4.

**Fig 5:**
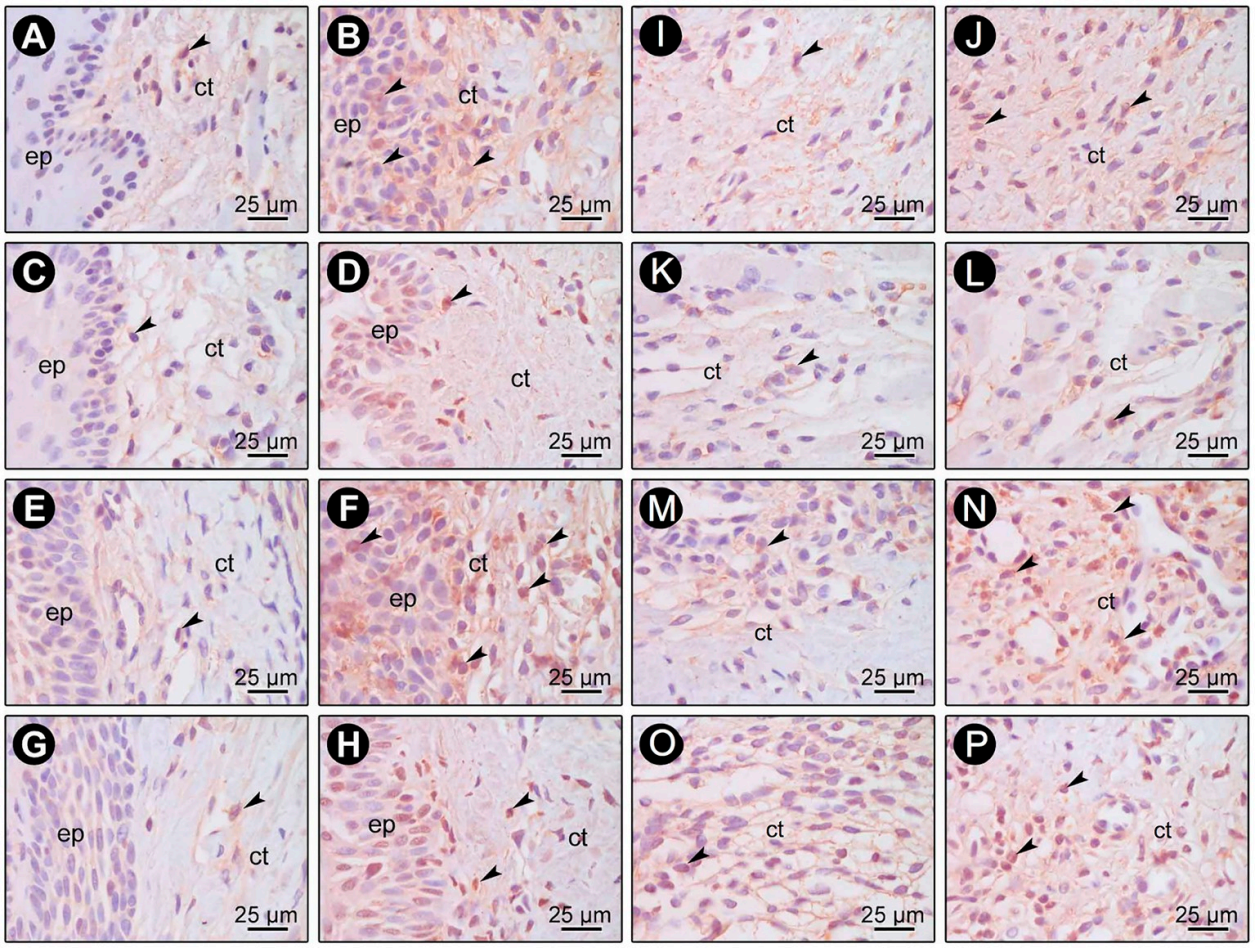
Photomicrographs showing BD-3 (A-H) and TLR4 (I-P) immunolabeling in gingival biopsies at baseline (A, C, E, G, I, K, M, O) and 30 days (B, D, F, H, J, L, N, P) from healthy (A-D, I-L) and diseased (E-H, M-P) sites in Test (A, B, E, F, I, J, M, N) and Control (C, D, G, H, K, L, O, P) groups. Arrows = BD-3 and TLR4-positive cells; ep = Epithelial tissue; ct = Connective tissue. Scale = 25 μm. Counterstaining: Hematoxylin.

**Fig 6:**
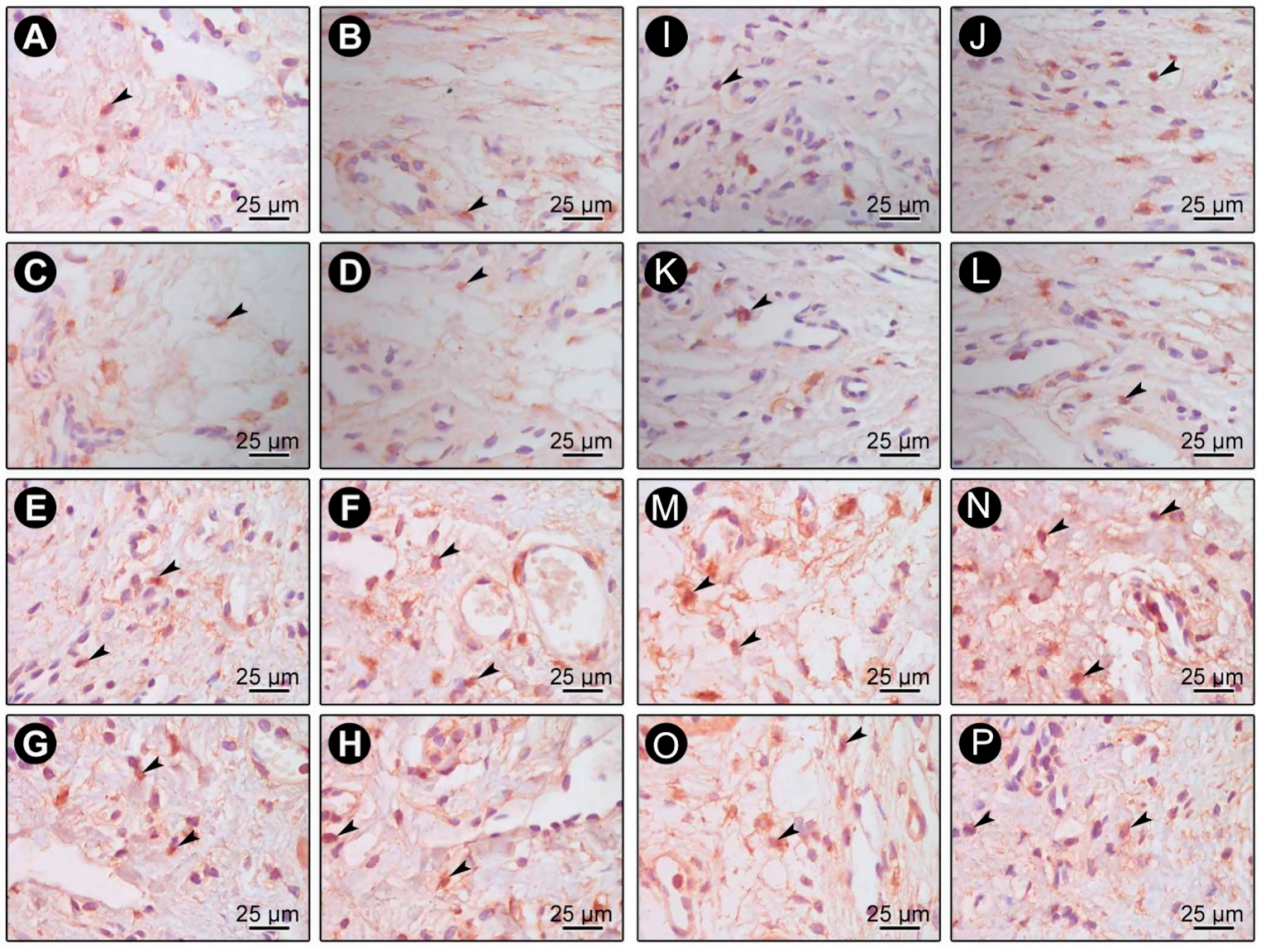
Photomicrographs showing CD-57 (A-H) and CD-4 (I-P) immunolabeling in gingival biopsies (connective tissue) at baseline (A, C, E, G, I, K, M, O) and 30 days (B, D, F, H, J, L, N, P) from healthy (A-D, I-L) and diseased (E-H, M-P) sites in Test (A, B, E, F, I, J, M, N) and Control (C, D, G, H, K, L, O, P) groups. Arrows = CD-57 and CD-4 positive cells. Scale = 25 μm. Counterstaining: Hematoxylin.

The immunohistochemical technique was highly specific for the detection of BD-3 (Fig 5A–5H), TLR4 (Fig 5I–5P), CD-57 (Fig 6A–6H) and CD-4 (Fig 6I–6P). No immunoreactivity was observed in the negative controls of immunohistochemical reactions. Immunolabeling stains were defined as dark brown and were circumscribed to cytoplasms and, to a lesser extent, to extracellular matrices. Immunolabeling of BD-3 was predominantly present in keratinocytes of epithelial tissue and in fibroblasts of connective tissue. Immunolabeling of TLR4 was predominantly present in cells of connective tissue. Immunolabeling of CD-4 and CD-57 was present mainly in inflammatory and macrophage-like cells.

Only the healthy sites of patients treated with probiotics exhibited significantly higher BD-3 and TLR4 immunoreactivity (Fig 3) at 30 days when compared to baseline (*p<0*.05). At diseased sites, there was significantly higher BD-3 and TLR4 immunoreactivity in both groups at 30 days when compared to baseline (*p<0*.05) (Fig 3). The Test group exhibited significantly higher BD-3 and TLR4 expressions on diseased sites at 30 days when compared to Control group (*p<0*.05) (Fig 3). In CD-57 analysis, there were no significant differences in intra-groups or inter-groups comparisons in healthy or diseased sites (Fig 4). No inter or intra-groups differences in the expression of CD-4 in healthy sites were observed (Fig 4). Considering diseased sites, only Test group showed a significantly greater immunoreactivity pattern of CD-4 at 30 days when compared with baseline (*p<0*.05) (Fig 4). At 30 days, CD-4 immunoreactivity pattern in diseased sites of Test group was also greater (*p<0*.05) than that of the Control group (Fig 4).

### *In vitro* assay of the adhesion of *B*. *lactis* HN019 and *P*. *gingivalis* to BEC

**Fig 7:**
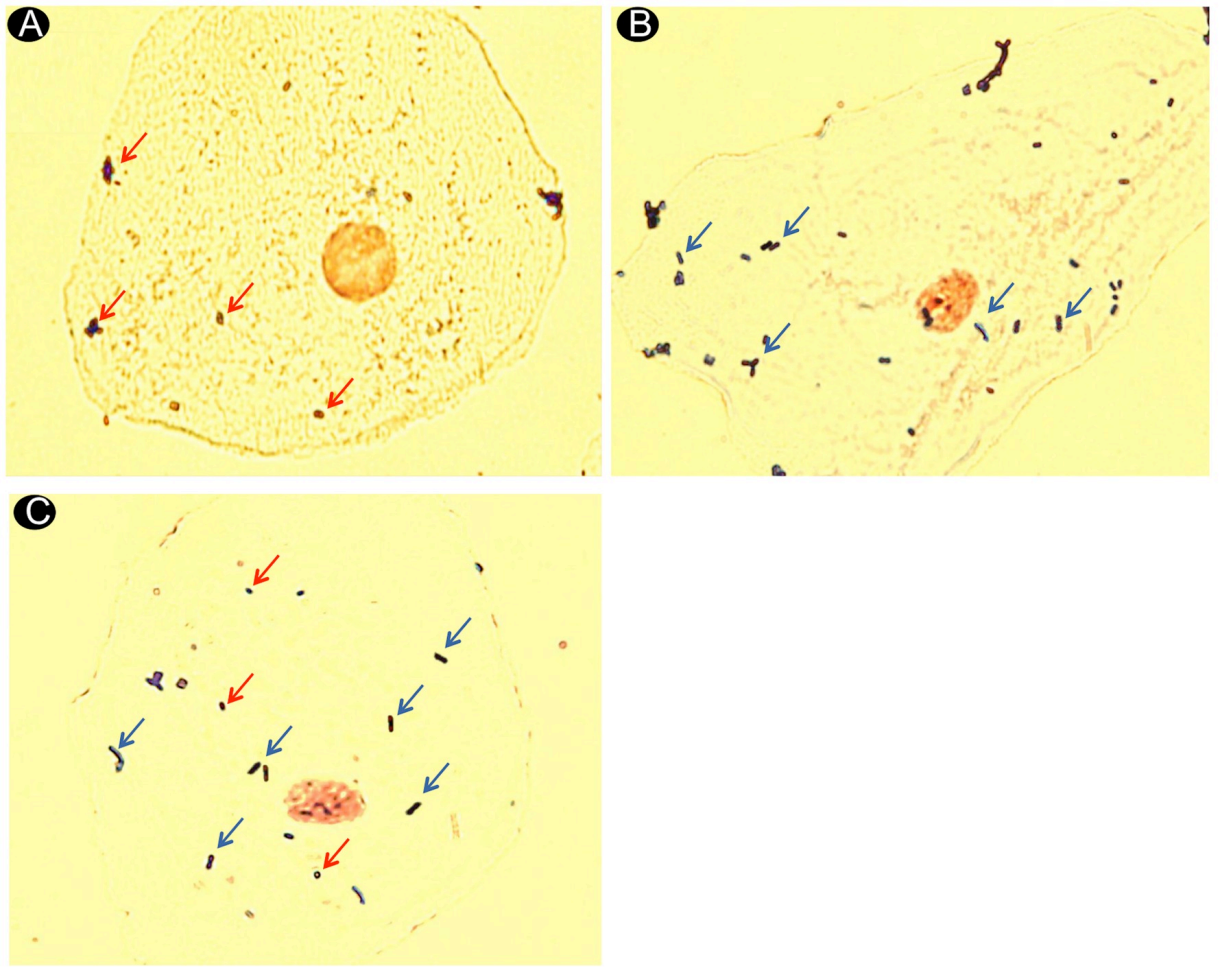
Photomicrographs showing buccal epithelial cells in adhesion assays using crystal violet and safranin. (**A**) *P*. *gingivalis* alone attached in a buccal cell (original magnification x100). (**B**)–*B*. *lactis* HN019 alone attached in a buccal cell (original magnification x100). (**C**)–Mixture of *B*. *lactis* HN019 with *P*. *gingivalis* attached in a buccal cell (original magnification x100). Red arrows indicate *P*. *gingivalis* (Gram-negative bacteria with shorter rods or coccobacilli morphology). Blue arrows indicate *Bifidobacterium* (Gram-positive bacteria with long bacilli morphology).

A background of approximately 13 indigenous bacteria/cells were attached to washed and untreated BEC. Table 2 shows the mean adhesion of *B*. *lactis* HN019 and *P*. *gingivalis* to BEC alone and combined. There was a lower mean adhesion of *P*. *gingivalis* combined with *B*. *lactis* HN019 when compared to the mean adhesion of *P*. *gingivalis* alone *(p<0*.*05)*. Similarly, the adhesion of *B*. *lactis* HN019 was influenced by the presence of *P*. *gingivalis* (Fig 7).

**Table 2:** Adhesion of *B*. *lactis* HN019 and *P*. *gingivalis* to buccal epithelial cells. Mean ± standard deviation of the bacteria counts.

Strains—Group	Mean ± SD
Counts of *B*. *lactis* HN019 alone–Group *B*. *lactis* HN019 alone	16.57 ± 16.21
Counts of *P*. *gingivalis* alone–Group *P*. *gingivalis* alone	7.21 ± 3.88
Counts of *B*. *lactis* HN019 –Group *B*. *lactis* HN019 mixed with *P*. *gingivalis*	6.00 ± 12.66*
Counts of *P*. *gingivalis*–Group *B*. *lactis* HN019 mixed with *P*. *gingivalis*	4.63 ± 6.48*^#^

*Significant different when compared with Group *B*. *lactis* HN019 alone.

^#^ Significant different when compared with Group *P*. *gingivalis* alone.

Binding to buccal epithelial cells = bacteria/cell–bacteria/cell in control (indigenous bacteria).

### *In vitro* analysis of *B*. *lactis* HN019 antimicrobial activity

**Fig 8:**
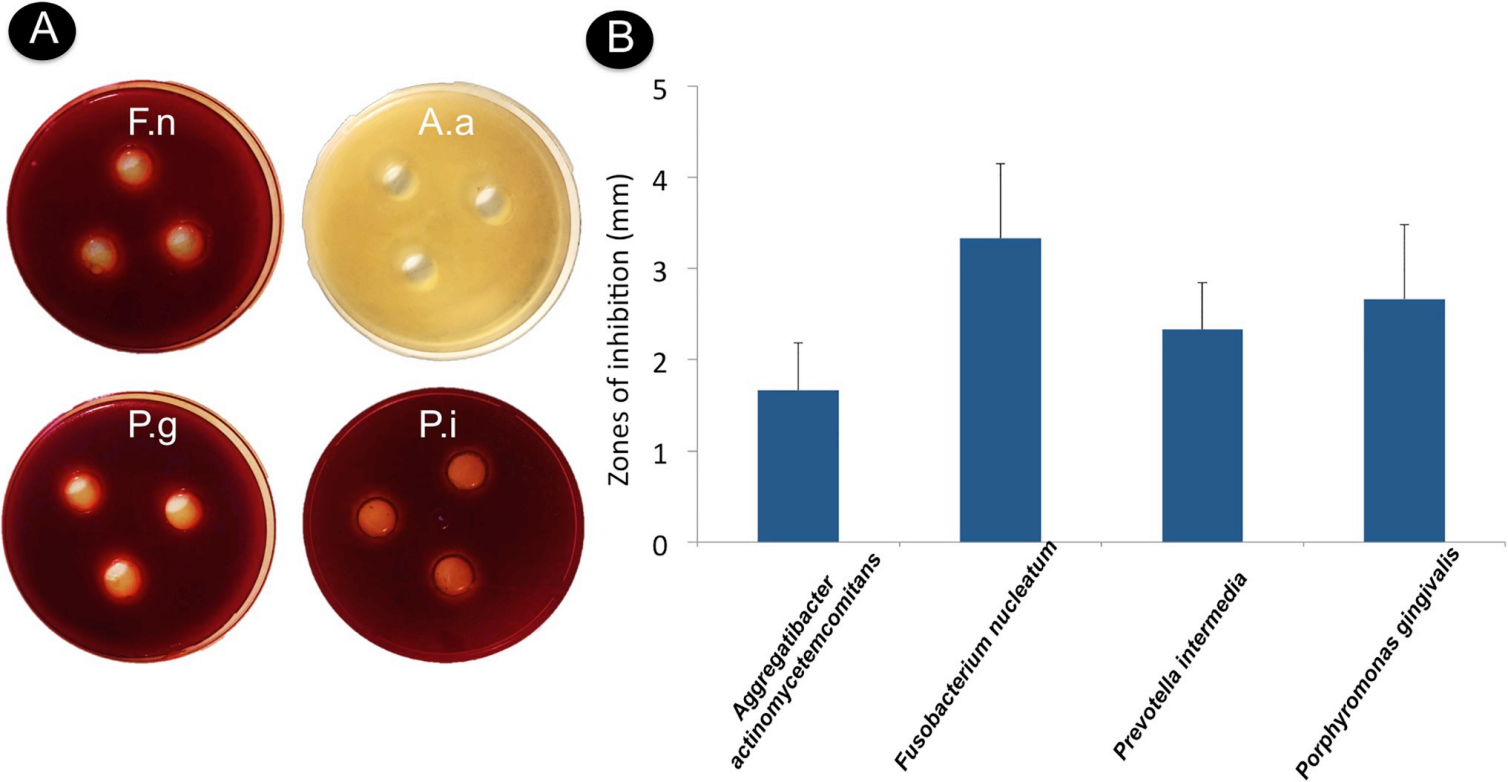
Growth inhibition of *Aggregatibacter actinomycetemcomitans*, *Porphyromonas gingivalis*, *Prevotella intermedia*, and *Fusobacterium nucleatum* by *Bifidobacterium animalis subs*. *Lactis* HN019 (agar diffusion method). (**A**) Clear areas around the wells indicate zones of inhibition. (**B**) Means and standard deviations of the zone of inhibition observed in sensitivity of different periodontopathogens to *B*. *lactis* HN019.

Means and standard deviations (mm) of the zones of inhibition obtained for the sensitivity of different periodontopathogens to *B*. *lactis* HN019 are displayed in Fig 8. The probiotic strain inhibited the growth of all periodontopathogens assessed.

## Discussion

This double-blind randomized controlled trial assessed clinical and immunological effects of *B*. *lactis* HN019 during non-surgical periodontal therapy in individuals with GCP. The findings demonstrate that probiotic therapy improved plaque control, reduced BOMP, and increased BD-3, TLR4 and CD-4 expressions in periodontal tissues. *B*. *lactis* HN019 also inhibited the growth of periodontopathogens in *in vitro* tests and reduced the adhesion of *P*. *gingivalis* to BEC.

In the present study, the Test group had significantly lower PI than the Control group at 30 days. Some previous studies have demonstrated that Bifidobacterium spp. and Lactobacillus spp. could quantitatively influence oral biofilm formation [19, 20]. Some probiotic strains may form biofilm on hard and soft tissues, preventing the adhesion of pathogenic bacteria, and they may also change the protein composition of the acquired salivary pellicle by binding and/or degradation of salivary proteins [22].

Bacterial coaggregation mechanisms are also likely to affect the quantity and quality of oral biofilm. The probiotic strain used in the present study can perfectly adhere to subgingival biofilms containing *P*. *gingivalis*, *Actinomyces naeslundii*, and *F*. *nucleatum* [33] and can coaggregate with *F*. *nucleatum* [22]. The latter act as an actual “biological bridge” in oral biofilm formation, as they adhere to virtually all oral bacteria [22]. The possible coaggregation of Bifidobacterium with *F*. *nucleatum* may have reduced the number of binding sites for other bacteria, affecting the quality and quantity of biofilm in patients from the Test group in the present study. Moreover, *B*. *lactis* HN019 may have directly inhibited the growth of different periodontopathogens. Previous *in vitro* studies demonstrated that *Bifidobacterium* could inhibit the growth of periodontopathogens [32, 33]. In the present study, *B*. *lactis* HN019 showed antimicrobial potential against *P*. *gingivalis*, *P*. *intermedia*, *F*. *nucleatum*, and *A*. *actinomycetemcomitans* in *in vitro* sensitivity tests.

Probiotic therapy, even when administered for a short time period, seems to promote remarkable resilience of the oral microbiome to risk factors for periodontitis, such as bacterial plaque accumulation [34]. Notwithstanding a PI similar to that of the Control group at 90 days, the Test group had a lower BOMP, which indicates a possible delayed effect of probiotic therapy on gingival inflammation. Lee et al. (2015) showed that *Lactobacillus brevis* lozenges significantly delayed the onset of gingival inflammation in healthy individuals who refrained from oral hygiene [35].

There are at least two ways to explain the lower gingival inflammation observed in patients from the Test group in the present study. The first is related to the possible competition between probiotic bacteria and periodontopathogens for binding sites. Mendi et al. (2016) conducted an *in vitro* study and demonstrated that *Lactobacillus rhamnosus* can adhere to mesenchymal stem cells in the gingiva, possibly protecting them from being colonized by *P*. *gingivalis* [36]. In the *in vitro* assays performed in this study, *B*. *lactis* HN019 was able to adhere to BEC and could reduce the adhesion of *P*. *gingivalis* to these cells. This is the first study to assess the potential adhesion of *B*. *lactis* HN019 to BEC. The mean adhesion observed in the present study was similar to the one found by Haukioja et al. (2006) for *Lactobacillus rhamnosus* GG in BEC [22]. However, it is important to emphasize that this study used only one volunteer for collection of BEC. Considering that adhesion could vary in different individuals, future studies should consider this. The second explanation could be related to a possible interference of probiotic bacteria in the expression of markers involved in regulating the immunocompetence of the epithelial barrier. The gingival biopsies of diseased sites showed an increase in BD-3 expression in Test group. A previous study showed that *B*. *lactis* HN019 also increased BD-3 expression in periodontal tissues and reduced periodontal inflammation in rats with experimental periodontitis [29]. BD are antimicrobial peptides that play an important role in epithelial innate immunity and their differential expressions are associated with periodontal diseases [37]. Among BDs, BD-3 is differentially induced by infectious or inflammatory stimuli [38–40]. Some studies [41–43] demonstrated that inflamed periodontal sites have lower BD-3 levels than do healthy sites. In the present study, Test group also exhibited higher TLR4 expression than Control group. TLR4 is a recognition receptor that plays a crucial role in the host’s innate response, as it helps identify lipopolysaccharides (LPS) of gram-negative bacteria [44]. The administration of *Lactobacillus casei* CRL 431 in healthy mice increased the number of TLR4-positive cells when compared to animals that were not treated with the probiotic. This finding, according to the authors, could indicate some improvement in physiological surveillance mechanisms against pathogenic bacteria [45].

In this study, gingival biopsies of diseased sites presented greater expressions of CD-57 than the ones of healthy sites. Natural killer (NK) cells, which are CD-57-positive, are lymphocytes that differ from B and T cells in phenotype and function. The response of NK cells to pathogens and tumoral cells is almost immediate and occurs before the development of the adaptive immune response [46]. Kopp (1988) observed an increase in NK cells in the gingiva of patients with chronic periodontitis when compared with the gingiva of healthy subjects [47]. In the present study, there were no significant differences in CD-57 immunolabeling pattern between Test and Control groups, although there was less marginal gingival bleeding in Test group. In fact, there are controversies in the literature regarding the relation between NK cells and the severity of periodontal disease. While several studies demonstrated some correlation between periodontal status and the number and phenotype of NK cells, others did not show any relation between them [47–51].

Besides innate immunity, cells of the adaptive immunity act in the pathogenesis of periodontal diseases, specially CD4+ and CD8+ T cells (T-helper (Th) cells). In the present study, diseased sites of patients of Test group presented greater expression of CD4+ cells than the ones of Control group. In fact, probiotic therapy may influence the expression of CD4+ cells. Probiotic administration increased CD4+ cells in the lamina propria of the small intestine of mice that were exposed to an experimental model of stress induced by food and mobility restriction [52]. Daily consumption of probiotics over a prolonged period of time may improve CD4+ count in people living with HIV [53]. It is important to emphasize that, in the pathogenesis of periodontal diseases, CD4+ cells function over other cell types can be dual depending on the cytokines produced [54]. In the present study, it is likely that CD4+ cells of Test group presented a phenotype related to the production of anti-inflammatory cytokines. Invernici et al. (2018) demonstrated that patients treated with *B*. *lactis* HN019 presented lower proinflammatory cytokine levels in gingival crevicular fluid than the patients not treated [30].

IgA is an important defense factor found in the saliva and it influences the oral microbiota, interfering with bacterial adhesion and metabolism [55]. The probiotic therapy used in the present study did not change IgA levels in the saliva. Studies on immunoregulatory effects of probiotic bacteria involving IgA have shown contradictory and inconclusive outcomes [56]. While several studies showed that probiotic bacteria of the genus *Lactobacillus* [57–61] did not increase IgA levels, other studies revealed high IgA concentrations [62–66] in patients treated with probiotic strains of the genera *Bifidobacterium*, *Lactobacillus*, or *Enterococcus*. There could be many reasons for the conflicting findings, such as heterogeneous research models, type of saliva, and analytical methodology [56]. Furthermore, the assessed saliva might not fully indicate the composition of salivary glands.

The short period of assessment is a limitation of this study. A long-term follow-up of the patients would be important to evaluate whether the additional effects obtained with probiotic therapy during non-surgical periodontal therapy will be sustained over time. It is also important that future investigations evaluate new methods of administration of *B*. *lactis* HN019 and other therapeutic regimens for the treatment of periodontitis patients. The effect of *B*. *lactis* HN019 on patients with gingivitis should also be investigated as well.

## Conclusions

In conclusion, the immunological and antimicrobial properties of *B*. *lactis* HN019 make it a potential probiotic to be used in non-surgical periodontal therapy of patients with GCP.

## Supporting information

S1 Checklist CONSORT checklist. (DOC)

S1 File Protocol study. (DOCX)

## References

[1] Marsh PD (2003) Are dental diseases examples of ecological catastrophes? Microbiology 149 (Pt 2):279–294. 10.1099/mic.0.26082-0 12624191

[2] Ximenez-Fyvie LA, Haffajee AD, Som S, Thompson M, Torresyap G, Socransky SS (2000) The effect of repeated professional supragingival plaque removal on the composition of the supra- and subgingival microbiota. Journal of clinical periodontology 27 (9):637–647 10.1034/j.1600-051x.2000.027009637.x 10983597

[3] Shibli JA, Melo L, Ferrari DS, Figueiredo LC, Faveri M, Feres M (2008) Composition of supra- and subgingival biofilm of subjects with healthy and diseased implants. Clinical oral implants research 19 (10):975–982. 10.1111/j.1600-0501.2008.01566.x 18828812

[4] Haffajee AD, Socransky SS, Patel MR, Song X (2008) Microbial complexes in supragingival plaque. Oral microbiology and immunology 23 (3):196–205. 10.1111/j.1399-302X.2007.00411.x 18402605

[5] Sanz-Sanchez I, Ortiz-Vigon A, Herrera D, Sanz M (2016) Microbiological effects and recolonization patterns after adjunctive subgingival debridement with Er:YAG laser. Clinical oral investigations 20 (6):1253–1261. 10.1007/s00784-015-1617-y 26419675

[6] Lindhe J, Westfelt E, Nyman S, Socransky SS, Heijl L, Bratthall G (1982) Healing following surgical/non-surgical treatment of periodontal disease. A clinical study. Journal of clinical periodontology 9 (2):115–128 10.1111/j.1600-051x.1982.tb01227.x 7042768

[7] Magnusson I, Lindhe J, Yoneyama T, Liljenberg B (1984) Recolonization of a subgingival microbiota following scaling in deep pockets. Journal of clinical periodontology 11 (3):193–207 10.1111/j.1600-051x.1984.tb01323.x 6368611

[8] Mark Bartold P, Van Dyke TE (2017). Host modulation: controlling the inflammation to control the infection. Periodontology 2000 75 (1):317‐329. 10.1111/prd.12169 28758299

[9] FAOlWHO J (2002) Working Group Report on Drafting Guidelines for the Evaluation of Probiotics in Food London. Ontario, Canada

[10] de Vrese M, Schrezenmeir J (2008) Probiotics, prebiotics, and synbiotics. Advances in biochemical engineering/biotechnology 111:1–66. 10.1007/10_2008_097 18461293

[11] Teughels W, Durukan A, Ozcelik O, Pauwels M, Quirynen M, Haytac MC (2013) Clinical and microbiological effects of Lactobacillus reuteri probiotics in the treatment of chronic periodontitis: a randomized placebo-controlled study. Journal of clinical periodontology 40 (11):1025–1035. 10.1111/jcpe.12155 24164569 PMC3908359

[12] Krasse P, Carlsson B, Dahl C, Paulsson A, Nilsson A, Sinkiewicz G (2006) Decreased gum bleeding and reduced gingivitis by the probiotic Lactobacillus reuteri. Swedish dental journal 30 (2):55–60 16878680

[13] Riccia DN, Bizzini F, Perilli MG, Polimeni A, Trinchieri V, Amicosante G, et al (2007) Anti-inflammatory effects of Lactobacillus brevis (CD2) on periodontal disease. Oral diseases 13 (4):376–385. 10.1111/j.1601-0825.2006.01291.x 17577323

[14] Twetman S, Derawi B, Keller M, Ekstrand K, Yucel-Lindberg T, Stecksen-Blicks C (2009) Short-term effect of chewing gums containing probiotic Lactobacillus reuteri on the levels of inflammatory mediators in gingival crevicular fluid. Acta odontologica Scandinavica 67 (1):19–24. 10.1080/00016350802516170 18985460

[15] Stensson M, Koch G, Coric S, Abrahamsson TR, Jenmalm MC, Birkhed D, et al (2014) Oral administration of Lactobacillus reuteri during the first year of life reduces caries prevalence in the primary dentition at 9 years of age. Caries research 48 (2):111–117. 10.1159/000354412 24296746

[16] Tekce M, Ince G, Gursoy H, Dirikan Ipci S, Cakar G, Kadir T, et al (2015) Clinical and microbiological effects of probiotic lozenges in the treatment of chronic periodontitis: a 1-year follow-up study. Journal of clinical periodontology 42 (4):363–372. 10.1111/jcpe.12387 25728888

[17] Ince G, Gursoy H, Ipci SD, Cakar G, Emekli-Alturfan E, Yilmaz S (2015) Clinical and Biochemical Evaluation of Lozenges Containing Lactobacillus reuteri as an Adjunct to Non-Surgical Periodontal Therapy in Chronic Periodontitis. Journal of periodontology 86 (6):746–754. 10.1902/jop.2015.140612 25741580

[18] Szkaradkiewicz AK, Stopa J, Karpiński TM (2014) Effect of oral administration involving a probiotic strain of Lactobacillus reuteri on pro-inflammatory cytokine response in patients with chronic periodontitis. Archivum immunologiae et therapiae experimentalis 62 (6):495–500. 10.1007/s00005-014-0277-y 24509697 PMC4244533

[19] Kuru BE, Laleman I, Yalnizoglu T, Kuru L, Teughels W (2017) The Influence of a Bifidobacterium animalis Probiotic on Gingival Health: A Randomized Controlled Clinical Trial. Journal of periodontology 88 (11):1115–1123. 10.1902/jop.2017.170213 28753102

[20] Penala S, Kalakonda B, Pathakota KR, Jayakumar A, Koppolu P, Lakshmi BV, et al (2016) Efficacy of local use of probiotics as an adjunct to scaling and root planing in chronic periodontitis and halitosis: A randomized controlled trial. Journal of research in pharmacy practice 5 (2):86–93. 10.4103/2279-042X.179568 27162801 PMC4843589

[21] Nissen L, Sgorbati B, Biavati B, Belibasakis GN (2014) Lactobacillus salivarius and L. gasseri down-regulate Aggregatibacter actinomycetemcomitans exotoxins expression. Annals of microbiology 64:611–617. 10.1007/s13213-013-0694-x 24860281 PMC4028514

[22] Haukioja A, Yli-Knuuttila H, Loimaranta V, Kari K, Ouwehand AC, Meurman JH, et al (2006) Oral adhesion and survival of probiotic and other lactobacilli and bifidobacteria in vitro. Oral microbiology and immunology 21 (5):326–332. 10.1111/j.1399-302X.2006.00299.x 16922933

[23] Corthesy B, Gaskins HR, Mercenier A (2007) Cross-talk between probiotic bacteria and the host immune system. The Journal of nutrition 137 (3 Suppl 2):781S–790S 10.1093/jn/137.3.781S 17311975

[24] Menard S, Candalh C, Bambou JC, Terpend K, Cerf-Bensussan N, Heyman M (2004) Lactic acid bacteria secrete metabolites retaining anti-inflammatory properties after intestinal transport. Gut 53 (6):821–828 10.1136/gut.2003.026252 15138208 PMC1774064

[25] Wilson M (2005) Microbial inhabitants of humans: their ecology and role in health and disease. Cambridge University Press.

[26] Becker MR, Paster BJ, Leys EJ, Moeschberger ML, Kenyon SG, Galvin JL, et al (2002) Molecular analysis of bacterial species associated with childhood caries. Journal of clinical microbiology 40 (3):1001–1009 10.1128/jcm.40.3.1001-1009.2002 11880430 PMC120252

[27] Fuller R, Gibson GR (1997) Modification of the intestinal microflora using probiotics and prebiotics. Scandinavian journal of gastroenterology Supplement 222:28–31. 10.1080/00365521.1997.11720714 9145443

[28] Ricoldi MST, Furlaneto FAC, Oliveira LFF, Teixeira GC, Pischiotini JP, Moreira ALG, et al (2017) Effects of the probiotic Bifidobacterium animalis subsp. lactis on the non-surgical treatment of periodontitis. A histomorphometric, microtomographic and immunohistochemical study in rats. PloS one 12 (6):e0179946 10.1371/journal.pone.0179946 28662142 PMC5491108

[29] Oliveira LF, Salvador SL, Silva PH, Furlaneto FA, Figueiredo L, Casarin R, et al (2017) Benefits of Bifidobacterium animalis subsp. lactis Probiotic in Experimental Periodontitis. Journal of periodontology 88 (2):197–208. 10.1902/jop.2016.160217 27660886

[30] Invernici MM, Salvador SL, Silva PHF, Soares MSM, Casarin R, Palioto DB, et al (2018) Effects of Bifidobacterium probiotic on the treatment of chronic periodontitis: A randomized clinical trial. Journal of clinical periodontology 45 (10):1198–1210. 10.1111/jcpe.12995 30076613 PMC6221043

[31] Armitage GC (1999) Development of a classification system for periodontal diseases and conditions. Annals of periodontology 4 (1):1–6. 10.1902/annals.1999.4.1.1 10863370

[32] Zhu Y, Xiao L, Shen D, Hao Y (2010) Competition between yogurt probiotics and periodontal pathogens in vitro. Acta odontologica Scandinavica 68 (5):261–268. 10.3109/00016357.2010.492235 20491536

[33] Jasberg H, Soderling E, Endo A, Beighton D, Haukioja A (2016) Bifidobacteria inhibit the growth of Porphyromonas gingivalis but not of Streptococcus mutans in an in vitro biofilm model. European journal of oral sciences 124 (3):251–258. 10.1111/eos.12266 27061393

[34] Rosier BT, Marsh PD, Mira A (2017) Resilience of the Oral Microbiota in Health: Mechanisms That Prevent Dysbiosis. Journal of dental research:22034517742139. 10.1177/0022034517742139 29195050

[35] Lee JK, Kim SJ, Ko SH, Ouwehand AC, Ma DS (2015) Modulation of the host response by probiotic Lactobacillus brevis CD2 in experimental gingivitis. Oral diseases 21 (6):705–712. 10.1111/odi.12332 25720615

[36] Mendi A, Kose S, Uckan D, Akca G, Yilmaz D, Aral L, et al (2016) Lactobacillus rhamnosus could inhibit Porphyromonas gingivalis derived CXCL8 attenuation. Journal of applied oral science: revista FOB 24 (1):67–75. 10.1590/1678-775720150145 27008259 PMC4775012

[37] Wang P, Duan D, Zhou X, Li X, Yang J, Deng M, et al (2015) Relationship between expression of human gingival beta-defensins and levels of periodontopathogens in subgingival plaque. Journal of periodontal research 50 (1):113–122. 10.1111/jre.12187 24814979

[38] Krisanaprakornkit S, Kimball JR, Weinberg A, Darveau RP, Bainbridge BW, Dale BA (2000) Inducible expression of human beta-defensin 2 by Fusobacterium nucleatum in oral epithelial cells: multiple signaling pathways and role of commensal bacteria in innate immunity and the epithelial barrier. Infection and immunity 68 (5):2907–2915 10.1128/iai.68.5.2907-2915.2000 10768988 PMC97503

[39] Harder J, Bartels J, Christophers E, Schroder JM (2001) Isolation and characterization of human beta -defensin-3, a novel human inducible peptide antibiotic. The Journal of biological chemistry 276 (8):5707–5713. 10.1074/jbc.M008557200 11085990

[40] Jia HP, Schutte BC, Schudy A, Linzmeier R, Guthmiller JM, Johnson GK, et al (2001) Discovery of new human beta-defensins using a genomics-based approach. Gene 263 (1–2):211–218 10.1016/s0378-1119(00)00569-2 11223260

[41] Vardar-Sengul S, Demirci T, Sen BH, Erkizan V, Kurulgan E, Baylas H (2007) Human beta defensin-1 and -2 expression in the gingiva of patients with specific periodontal diseases. Journal of periodontal research 42 (5):429–437. 10.1111/j.1600-0765.2006.00964.x 17760820

[42] Bissell J, Joly S, Johnson GK, Organ CC, Dawson D, McCray PB Jr., et al (2004) Expression of beta-defensins in gingival health and in periodontal disease. Journal of oral pathology & medicine: official publication of the International Association of Oral Pathologists and the American Academy of Oral Pathology 33 (5):278–285. 10.1111/j.0904-2512.2004.00143.x 15078488

[43] Kuula H, Salo T, Pirila E, Hagstrom J, Luomanen M, Gutierrez-Fernandez A, et al (2008) Human beta-defensin-1 and -2 and matrix metalloproteinase-25 and -26 expression in chronic and aggressive periodontitis and in peri-implantitis. Archives of oral biology 53 (2):175–186. 10.1016/j.archoralbio.2007.09.010 17996844

[44] Medzhitov R, Preston-Hurlburt P, Janeway CA Jr. (1997) A human homologue of the Drosophila Toll protein signals activation of adaptive immunity. Nature 388 (6640):394–397. 10.1038/41131 9237759

[45] Castillo NA, Perdigon G, de Moreno de Leblanc A (2011) Oral administration of a probiotic Lactobacillus modulates cytokine production and TLR expression improving the immune response against Salmonella enterica serovar Typhimurium infection in mice. BMC microbiology 11:177 10.1186/1471-2180-11-177 21813005 PMC3173335

[46] Yokoyama WM, Kim S, French AR (2004) The dynamic life of natural killer cells. Annual review of immunology 22:405–29. 10.1146/annurev.immunol.22.012703.104711 15032583

[47] Kopp W (1988) Density and localization of lymphocytes with natural-killer (NK) cell activity in periodontal biopsy specimens from patients with severe periodontitis. Journal of clinical periodontology 15 (10):595–600. 10.1111/j.1600-051x.1988.tb02257.x 3058751

[48] Wynne SE, Walsh LJ, Seymour GJ, Powell RN (1996) In situ demonstration of natural killer (NK) cells in human gingival tissue. Journal of periodontology 57 (11):699–702. 3494114 10.1902/jop.1986.57.11.699

[49] Fujita S, Takahashi H, Okabe H, Ozaki Y, Hara Y, Kato I (1992) Distribution of natural killer cells in periodontal diseases: an immunohistochemical study. Journal of periodontology 63 (8):686–9. 10.1902/jop.1992.63.8.686 1507049

[50] Stelin S, Ramakrishan H, Talwar A, Arun KV, Kumar TS (2009) Immunohistological analysis of CD1a langerhans cells and CD57 natural killer cells in healthy and diseased human gingival tissue: A comparative study. Journal of indian society of periodontology 13 (3):150–154. 10.4103/0972-124X.60228 20379413 PMC2848786

[51] Cobb CM, Singla O, Feil PH, Theisen FC, Schultz RE (1989) Comparison of NK-cell (Leu-7+ and Leu-11b+) populations in clinically healthy gingiva, chronic gingivitis and chronic adult periodontitis. Journal of periodontal research 24 (1):1–7. 10.1111/j.1600-0765.1989.tb00851.x 2524565

[52] Palomar MM, Maldonado Galdeano C, Perdigón G (2014) Influence of a probiotic lactobacillus strain on the intestinal ecosystem in a stress model mouse. Brain, behavior, and immunity 35:77–85. 10.1016/j.bbi.2013.08.015 24016865

[53] Miller H, Ferris R, Phelps BR (2016) The effect of probiotics on CD4 counts among people living with HIV: a systematic review. Beneficial microbes 7 (3):345–351. 10.3920/BM2015.0163 27013461

[54] Romagnani S (1997) The Th1/Th2 paradigm. Immunology today 8 (6):263–266. 10.1016/s0167-5699(97)80019-9 9190109

[55] Patil PB, Patil BR (2011) Saliva: A diagnostic biomarker of periodontal diseases. Journal of Indian Society of Periodontology 15 (4):310 10.4103/0972-124X.92560 22368352 PMC3283925

[56] Jorgensen MR, Keller MK, Kragelund C, Hamberg K, Ericson D, Nielsen CH, et al (2016) Lactobacillus reuteri supplements do not affect salivary IgA or cytokine levels in healthy subjects: A randomized, double-blind, placebo-controlled, cross-over trial. Acta odontologica Scandinavica 74 (5):399–404. 10.3109/00016357.2016.1169439 27104984

[57] Paineau D, Carcano D, Leyer G, Darquy S, Alyanakian MA, Simoneau G, et al (2008) Effects of seven potential probiotic strains on specific immune responses in healthy adults: a double-blind, randomized, controlled trial. FEMS immunology and medical microbiology 53 (1):107–113. 10.1111/j.1574-695X.2008.00413.x 18422632

[58] Gleeson M, Bishop NC, Oliveira M, McCauley T, Tauler P, Lawrence C (2012) Effects of a Lactobacillus salivarius probiotic intervention on infection, cold symptom duration and severity, and mucosal immunity in endurance athletes. International journal of sport nutrition and exercise metabolism 22 (4):235–242 10.1123/ijsnem.22.4.235 22645171

[59] Kekkonen RA, Lummela N, Karjalainen H, Latvala S, Tynkkynen S, Jarvenpaa S, et al (2008) Probiotic intervention has strain-specific anti-inflammatory effects in healthy adults. World journal of gastroenterology 14 (13):2029–2036 10.3748/wjg.14.2029 18395902 PMC2701523

[60] Tiollier E, Chennaoui M, Gomez-Merino D, Drogou C, Filaire E, Guezennec CY (2007) Effect of a probiotics supplementation on respiratory infections and immune and hormonal parameters during intense military training. Military medicine 172 (9):1006–1011 10.7205/milmed.172.9.1006 17937368

[61] Dong H, Rowland I, Thomas LV, Yaqoob P (2013) Immunomodulatory effects of a probiotic drink containing Lactobacillus casei Shirota in healthy older volunteers. European journal of nutrition 52 (8):1853–1863. 10.1007/s00394-012-0487-1 23307112

[62] Surono IS, Koestomo FP, Novitasari N, Zakaria FR, Yulianasari, Koesnandar (2011) Novel probiotic Enterococcus faecium IS-27526 supplementation increased total salivary sIgA level and bodyweight of pre-school children: a pilot study. Anaerobe 17 (6):496–500. 10.1016/j.anaerobe.2011.06.003 21763445

[63] Rizzardini G, Eskesen D, Calder PC, Capetti A, Jespersen L, Clerici M (2012) Evaluation of the immune benefits of two probiotic strains Bifidobacterium animalis ssp. lactis, BB-12(R) and Lactobacillus paracasei ssp. paracasei, L. casei 431(R) in an influenza vaccination model: a randomised, double-blind, placebo-controlled study. The British journal of nutrition 107 (6):876–884. 10.1017/S000711451100420X 21899798

[64] Shimizu K, Sato H, Suga Y, Yamahira S, Toba M, Hamuro K, et al (2014) The effects of Lactobacillus pentosus strain b240 and appropriate physical training on salivary secretory IgA levels in elderly adults with low physical fitness: a randomized, double-blind, placebo-controlled trial. Journal of clinical biochemistry and nutrition 54 (1):61–66. 10.3164/jcbn.13-62 24426193 PMC3882487

[65] Kotani Y, Shinkai S, Okamatsu H, Toba M, Ogawa K, Yoshida H, et al (2010) Oral intake of Lactobacillus pentosus strain b240 accelerates salivary immunoglobulin A secretion in the elderly: A randomized, placebo-controlled, double-blind trial. Immunity & ageing: I & A 7:11 10.1186/1742-4933-7-11 20796295 PMC2936365

[66] Asama T, Arima TH, Gomi T, Keishi T, Tani H, Kimura Y, et al (2015) Lactobacillus kunkeei YB38 from honeybee products enhances IgA production in healthy adults. Journal of applied microbiology 119 (3):818–826. 10.1111/jam.12889 26121394

