# Supplementary material for: Bifidobacterium animalis subsp lactis HN019 presents antimicrobial potential against periodontopathogens and modulates the immunological response of oral mucosa in periodontitis patients
Source: PLoS One. 2020 Sep 22;15(9):e0238425. doi: 10.1371/journal.pone.0238425 (PMC7508403; doi:10.1371/journal.pone.0238425)
Supplement: S1 File — (DOCX) [file pone.0238425.s002.docx]

Clinical, immunological and microbiological effects of probiotic therapy as an adjuvant to the non-surgical treatment of Chronic Periodontitis.

**Abstract**

Whereas the two main strategies for the treatment of periodontal diseases (PD) are the elimination of specific pathogens and suppression of destructive host response, the use of probiotics indubitability opens a promising field to achieve these goals. The aim of this study is to evaluate the effect of the probiotic therapy (ProbT) as an adjunct to non-surgical periodontal treatment (nsPT) in patients diagnosed with Generalized Chronic Periodontitis (GCP). In a randomized double-blind and placebo-controlled clinical trial, 30 patients with a clinical diagnosis of GCP will be treated with ProbT associated with scaling and root planing (SRP) or SRP only. In ProbT, the commercial strain *Bifidobacterium animalis subsp. lactis* HN019 will be consumed once a day during 4 weeks immediately after the first session of mechanical instrumentation. All patients will be monitored for 90 days. Clinical assessments of plaque index, probing depth, relative clinical attachment level and bleeding on probing will be performed at baseline (pre-intervention period), 30 and 90 days after the nsPT. Subgingival plaque samples will be collected (at baseline and 30 and 90 days after the nsPT) and the counts of 40 subgingival species will be determined using DNA-DNA checkerboard hybridization. Gingival crevicular fluid samples will be collected (at baseline and 30 and 90 days after the nsTP) for evaluation of the volume of fluid (Periotron) and the levels of Interleukin-1 beta, Interleukin-8 and Interleukin-10 (Luminex). Saliva samples will be also collected in the same periods of time to determine the levels of Immunoglobulin A. Data obtained will be statistically analyzed.

Key-words: Chronic Periodontitis; Probiotic; Dental Scaling.

**Methods**

**1. Subject population, inclusion and exclusion criteria**

All patients will receive detailed information regarding the study (goals, benefits and risks) according to the Term of Consent. The experimental protocol will be subjected to approval by the University of Sao Paulo’s Ethics Committee in Clinical Research (School of Dentistry of Ribeirao Preto - University of Sao Paulo).

The sample size was determined using the software Graphpad Statemate 2.0 (GraphPad Software, Inc., San Diego, CA, USA). The ideal sample size to ensure an 80% power in the statistical analysis of the data obtained in this study was calculated considering the differences of means and standard deviations between the test and control groups of the study by Vivekananda et al. (2010). The α value was set at 0.05. The average dropout of patients in our previous studies (approximately 20%) was also considered to calculate the sample size. Thus, a sample size of 30 patients was considered appropriated for this study.

A total of 30 patients diagnosed with generalized chronic periodontitis, according to the current classification of the American Academy of Periodontology (ARMITAGE, 1999), will be recruited at the Periodontal Clinic of the School of Dentistry of Ribeirao Preto - University of Sao Paulo (Ribeirao Preto, Sao Paulo, SP, Brazil). The inclusion criteria for the present study will be: >30 years of age, presence of at least 15 natural teeth (excluding third molars and teeth indicated for extraction), 30% or more of the sites with probing depth (PD) ≥ 4 mm, clinical attachment level (CAL) ≥ 4 mm, presence of bleeding on probing and a minimum of six teeth with at least one site of each with CAL and PD ≥ 5 mm. All patients must present good general health. Exclusion criteria for the present study will be: cause-related periodontal therapy in the previous 6 months, antimicrobial therapy in the previous 6 months, systemic conditions that may influence the progression of periodontitis or the response to the treatment (i.e. diabetes mellitus, immunological disorders), pregnancy, smoking, extensive prosthetic appliances, need of prophylactic antibiotic therapy for routine dental procedures and long-term administration of anti-inflammatory medications.

**2. Experimental design and treatment protocols**

Before the study begins, the selected individuals will be identified by a numeric code and will receive instructions regarding oral hygiene as well as supragingival scaling in all teeth. According to a random numeric table generated by a computer software, the study coordinator will allocate each patient into one of the following groups: Control (Scaling and Root Planing – SRP; 18 patients) or Test (SRP + probiotic therapy). The subjects (Control and Test groups) will receive sachets containing 30 g of powdered milk. In the Test group, the sachets will present 10^9^ colony-forming units (CFUs) of *Bifidobacterium animalis subsp*. *lactis* HN019 per gram of powdered milk. Individuals will be instructed (immediately after the first session of mechanical instrumentation) to consume the powdered milk once a day for 4 weeks by dissolving the contents of the sachets into 250 mL of water immediately before ingestion (GOPAL et al. 2003). They will also be instructed not to consume other probiotic product during the study. Clinical, immunological and microbiological parameters will be assessed at baseline (pre-intervention) and after completion of non-surgical periodontal therapy.

Periodontal clinical examination (pre- and post-intervention) will be conducted by a single trained and calibrated examiner, who will be blinded to the experimental groups of the study. The examiner will also be blinded to the microbiological and immunological evaluations. SRP procedures will be performed by another specialist in Periodontics, trained for these purposes, who will also be blinded to the experimental groups of the study.

The School of Pharmaceutical Sciences of the University of Sao Paulo (São Paulo, SP, Brazil) will prepare the probiotic and non-probiotic (placebo) powdered milk, which will be stored in identical sachets. The sachets will be sent to the coordinator of the study, who will mark the code number of each subject on a set of 28 sachets (amount to be consumed by each subject during 4 weeks), according to the experimental group assigned. The coded sachets will be given to the examiner, who will distribute them to the patients and will not have any access to information regarding the content of the sachets. In addition, the patients will be blinded to the content of the sachets and the treatment assignment during the study. The meaning of each code number will be revealed by the study coordinator only after conducting the statistical analysis of the experimental data.

The subjects will receive seven sachets (placebo or probiotic) by week. At the end of each week, they shall attend at the Periodontal Clinic of the School of Dentistry of Ribeirao Preto - University of Sao Paulo. During this visit, they shall bring the packs of sachets that were consumed during the week and then they will receive new sachets, sufficient for another week of consumption. At this visit, patients will respond a questionnaire about their perception of any side effect observed during the consumption of the dietary supplement. Two research assistants will conduct these procedures and they will be responsible for monitoring the patient's compliance in the consumption of sachets provided. These assistants will not be examiners or operators in this study.

**3. Clinical monitoring**

Each patient will be impressed with alginate in order to obtain models of the dental arches and elaborate a guide plate made of acetate. This plate will present grooves that will be used as references to standardize the insertion and tilt of the automated periodontal probe (Florida Probe System, Florida Probe Corporation, Gainesville, FL, USA). The visible plaque index for each patient, rated dichotomously (O'Leary et al. 1972), will be determined by the percentage of tooth surfaces with deposits of plaque stained with disclosing solution.

The following clinical periodontal parameters will be assessed at 6 sites of each tooth (mesio-buccal, buccal, disto-buccal, mesio-lingual, lingual and disto-lingual): (i) probing depth of periodontal pockets (mm): it will be measured from the gingival margin to the bottom of the pocket; (ii) relative clinical attachment level (mm): it will be measured as the distance from the occlusal surface of the guide plate to the bottom of the pocket; (iii) bleeding on probing, evaluated dichotomously (Ainamo and Bay, 1976): the presence of the bleeding will be considered positive when occurring within 20 seconds from the insertion of the probe for probing depth.

The clinical periodontal parameters and the plaque index of each patient will be recorded at baseline (pre-intervention), as well as +30 and +90 days after the non-surgical periodontal therapy.

**4. Examiner calibration**

The Kappa index will be used to evaluate the examiner calibration on clinical periodontal parameters collection in order to calculate the intra-examiner agreement. According to the World Health Organization (WHO) criteria for diagnosis, the acceptable Kappa index of agreement must be greater than or equal to 0.85 (WHO, 1997). This level of agreement will be used for calibration of the examiner in this project. Ten patients, each one showing at least two pairs of contralateral single-rooted teeth with PD ≥ 5 mm on interproximal sites, will be selected to calibrate the examiner. Each patient will be evaluated on two separate occasions 48 hours apart in order to obtain the intra-examiner reliability through the Kappa index.

**5. Immunological monitoring**

5.1. Gingival crevicular fluid sampling

At baseline and +30 and +90 days after the non-surgical periodontal therapy, GCF samples will be obtained from nine noncontiguous interproximal sites of each patient. These sites will be randomly selected, keeping a balance among the different quadrants involved and grouped according to the probing depth (PD) of the periodontal pocket during baseline. Therefore, 3 samples will be obtained for each one of the following categories: shallow (PD ≤ 3 mm), intermediary (PD = 4-6 mm) and deep (PD ≥ 7 mm). The supragingival plaque from selected teeth will be removed and the sites will be carefully dried with air jets, and subsequently isolated with sterile cotton rolls. Samples of GCF will be obtained with papers strips (Periopaper® -Oralflow Inc., Amityville, NY, USA). The papers strips will be gently inserted into the orifice of the periodontal pocket, remaining 30 seconds subgingivally. The amount of GCF absorbed will be determined by an electronic measurer of humid mass (Periotron® -Oralflow Inc., Amityville, NY, USA). Samples will be placed in sterile Eppendorf tubes stored at - 80°C for cytokines (IL-1β, IL-8 and IL-10) quantification (pg/μl) at Genese Laboratory (Genese Produtos Diagnosticos LTDA, Sao Paulo, SP, Brazil). Cytokines levels will be determined using a three-plex Millipore kit (Millipore Corporation, Billerica, MA, USA) and the Luminex 100TM system (Luminex, MiraiBio, Alameda, CA, USA).

5.2. Saliva sampling

At baseline, as well as +30 and +90 days after the non-surgical periodontal therapy, non-stimulated saliva will be collected in the morning, with the patient fasting for a minimum of 8 hours. Subjects will be informed that they shall avoid making movements with their tongue, cheeks or lips during the saliva collection. Two minutes after the instructions, participants will eliminate saliva on the spittoon in order to throw away the initial sediment. From this moment on, the collection will start at point zero. 3-minute cycles will be made, in which patients will keep saliva in the oral cavity. At the end of this period, they will throw away the residual saliva in a Falcon polypropylene tube of 50 mL. The action will be repeated 2 more times and the total collection time will be 9 minutes. Saliva samples will then be separated with manual micropipette in graduated Eppendorf tubes (1.5 mL) and immediately transported to the Laboratory of Clinical Analyses from the Hospital of the Medicine School of Ribeirao Preto-USP (USP-HCFMRP) to determine the levels of Immunoglobulin A (IgA).

**6. Microbiological Monitoring**

At baseline and +30 and +90 days after the non-surgical periodontal therapy, samples of subgingival plaque will be obtained from nine noncontiguous interproximal sites of each patient. These sites will be randomly selected, keeping a balance among the different quadrants involved and grouped according to the PD of the periodontal pocket during baseline. Therefore, 3 samples will be obtained for each one of the following categories: shallow (PD ≤ 3 mm), intermediary (PD = 4-6 mm) and deep (PD ≥ 7 mm). Samples will be individually analyzed for their content of 40 subgingival bacterial species using the checkerboard DNA–DNA hybridization technique (Socransky et al., 2004a; Socransky et al., 2004b). The selected teeth will be isolated with sterile cotton rolls and dried with air jets. Then, the supragingival plaque will be carefully removed using a sterile curette. Another sterile curette will be used to collect the subgingival plaque, starting from the bottom of the periodontal pocket to its coronal portion. The samples will be stored in sterilized Eppendorf tubes and will be processed at the Microbiology Laboratory of the Guarulhos University (UNG, Guarulhos, SP, Brazil).

**7. Non-surgical periodontal treatment**

Seven days prior to the non-surgical periodontal therapy, periapical radiographs will be taken from the whole mouth of all patients. They will be set in an oral hygiene program (OHP) according to their specific needs. In this program, patients will be instructed about an effective self-performed plaque control, including information about the Bass Technique (Bass, 1954) and interproximal cleaning with dental floss and interdental brushes. They will be also motivated to brush the dorsal surface of the tongue once a day and will receive a dentifrice that shall be used throughout the experimental period (Colgate Total®, Anakol Ind. Com Ltda - Brazil's Kolynos - Colgate Palmolive Co., Sao Bernardo do Campo, SP, Brazil). After the OHP, subjects will undergo the assessment of clinical periodontal parameters previously described and the collection of subgingival plaque and GCF in selected sites (baseline) will be performed. Shortly, patients will receive supragingival scaling and coronal polishing with prophy cup on all the teeth present in the oral cavity.

The non-surgical periodontal therapy will initiate 7 days after the OHP and initial supragingival scaling. Within 24 hours, a specialist in Periodontics will perform supra and subgingival scaling and root planing of all teeth with periodontal involvement, using hand (Gracey Curettes, Hu-Friedy, Chicago, IL, USA) and ultrasonic instruments. The instrumentation will be performed on each quadrant until achievement of an adequate cleaning and root planing, which will be verified with a dental explorer. Individuals will receive professional prophylaxis biweekly during three months after the end of the nonsurgical periodontal therapy. On biweekly follow-up visits, patient's cooperation will be monitored by verifying the status of oral hygiene.

**8. Statistical analysis**

The normality and homoscedasticity of the data obtained will be checked. Comparisons within groups and among groups at different time intervals will be performed through parametric or non-parametric appropriate tests. The significance level will be set at 5% in all tests. All calculations will be performed by SPSS software (SPSS, Chicago IL, USA).
